# Front-to-Side Hard and Soft Biometrics for Augmented Zero-Shot Side Face Recognition

**DOI:** 10.3390/s25061638

**Published:** 2025-03-07

**Authors:** Ahuod Hameed Alsubhi, Emad Sami Jaha

**Affiliations:** Department of Computer Science, Faculty of Computing and Information Technology, King Abdulaziz University, Jeddah 21589, Saudi Arabia; ejaha@kau.edu.sa

**Keywords:** human identification, face recognition, side face recognition, front-to-side face, zero-shot, hard biometrics, soft biometrics, feature-level fusion, deep learning, augmentation

## Abstract

Face recognition is a fundamental and versatile technology widely used to identify individuals. The human face is a significant nonintrusive biometric modality, attracting numerous research studies. Still, much less focus has been on side-face views, with the majority merely or mainly concentrating on the frontal face. Despite offering fewer traits than the front viewpoint, the side viewpoint of the face is a crucial aspect of an individual’s identity and, in numerous cases, can be the only available information. Our research proposes new soft biometric traits based on the face anthropometric that can be invariantly extracted from the front and side face. We aim to extract and fuse them with vision-based deep features to augment zero-shot side face recognition. Our framework uses the person’s front face information solely for training, then uses their side face information as the only query for biometric matching and identification. For performance evaluation and comparison of the proposed approach, several feature-level fusion experiments were conducted on the CMU Multi-PIE dataset. Our results demonstrate that fusing the proposed face soft traits with the ResNet-50 deep features significantly improves performance. Furthermore, adding global soft biometrics to them improves the accuracy by up to 23%.

## 1. Introduction

Nowadays, among several active research domains, biometrics has emerged as an essential field rich in innovations, systems, and many investigations on different biometric modalities, such as face, ear, iris, voice, and fingerprint. A typical biometric system refers to the process of identifying or verifying individuals’ identities. Face recognition is a biometric technique that utilizes an individual’s distinctive facial features to identify or authenticate their identity. Face biometrics technology has expanded tremendously and rapidly in recent years, becoming useful in a wide range of applications, including security and access control (e.g., unlocking cellphones or protecting sensitive locations), user authentication (for online banking or social networking), and law enforcement (for example, identifying suspects or missing persons) [1].

**Figure 1:**
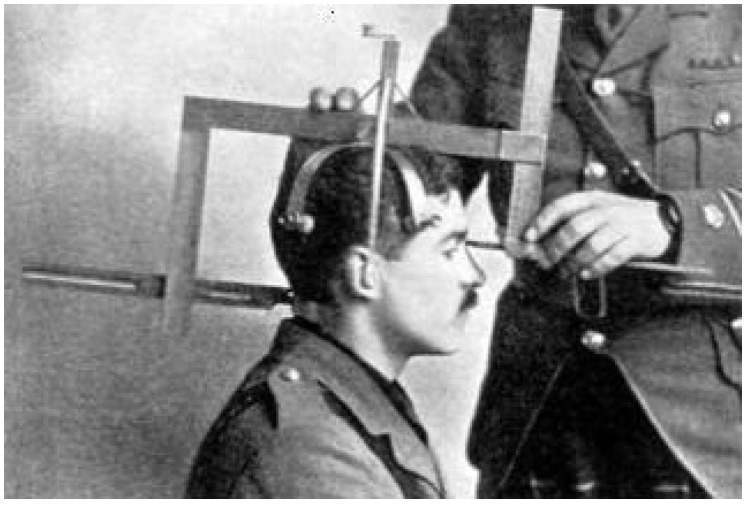
Alphonse Bertillon’s human head measurements.

Historically, the identification task is closely linked to human evolution, and prior to the development of involved modern technologies like computer vision and machine learning, early humans relied on vital formal and descriptive features of one another’s distinction. Such early enforced features were devoted to various scenarios for crime investigation, criminal records, and registration of civil and other identities. In 1879, Alphonse Bertillon created a system he hoped would address the deteriorating quality of record-keeping and the absence of trustworthy criminal identification mechanisms [2]. Figure 1 visually demonstrates Bertillon’s solution method, which involves taking a number of anthropometric measures with specially created calipers, gauges, and rulers on inflexible body parts, including height, head length, and head breadth [3].

The concept of “soft biometrics” describes the identification or verification of people using a new form of nontraditional semantic biometric traits. Unlike traditional biometrics like fingerprints or iris scans, soft biometrics rely on high-level semantically descriptive traits, such as namable information regarding a person’s appearance, behavior, or other features [4]. As the phrase implies, recognizing faces via soft biometrics can be achieved using semantic facial traits as soft biometric features, which are more general and possibly unique features of the face, such as age, gender, hair, etc. Human face analysis is considered a rich source of identity information, particularly the frontal face image, where all the features are ideally observable and representable. Additionally, the side view of the face is a key component of human identity [5], and it possesses many characteristics comprising more distinguishing features, such as the shape of the mouth, chin, and nose, which can often be more perceptible than in a frontal view.

Compared to frontal face image data, side face images earn much less attention in biometrics, and the existing research on side face recognition is still far less common than that on frontal face counterparts. Despite the advancement of the current face recognition systems, the identification of individuals based on their side view of the face remains insufficiently solved because of challenges such as pose variations [6]. Furthermore, current algorithms were mostly designed for front face recognition and excessively trained on frontal face images, where even in the most difficult cases, all facial components are adequately visible and usable (e.g., both the person’s eyes and face sides). However, they do not pay attention to and handle the recognition from the side viewpoint as excellently as the front of a face. That is because the frontal face viewpoint is considered the ideal and richest resource of biometric features, whereas some features are absent from the side face viewpoint.

In forensics and criminal investigations, the side-face viewpoint may often be crucial to identifying individuals. In many real-life situations, at hundreds of crime scenes, detectives or policemen may only have side-face surveillance images of a suspect or victim where many automated systems may fail to recognize them. Traditional face recognition algorithms have achieved high performance and made improvements over time; however, they still struggle to recognize side-face images due to several issues, including various positions, lighting, occlusion, and facial expressions [7,8]. Moreover, the lack of front-side invariant facial features in biometric systems makes side-face recognition further challenging [9].

Developing an adaptive biometric system that can recognize faces from both front and side (profile) viewpoints is crucial. Although the side viewpoint of the face offers fewer traits than the front viewpoint, it still comprises viable traits of the individual, and those can be the only available identity information in dozens of cases. Such challenging cases require a robust system that is somehow capable of identifying individuals even if it was trained only on front image data. This system should be able to exploit the only available unseen side face image as a query to identify. This paper investigates the feasibility of fusing a novel set of soft biometrics features with vision-based hard/traditional deep face features to augment side face recognition. Compared to hard/traditional techniques, one of the primary benefits of soft biometric features is their ability to adapt to environmental variations. They can additionally help improve recognition performance when combined with traditional face biometric traits. This paper’s primary contributions can be summarized as follows:

- Proposing a novel set of face soft biometrics that can be invariantly observed from the front and side face view.
- Fusing the proposed soft features with cited face hard biometrics for effective zero-shot front-to-side face recognition.
- Adopting a proper feature-level fusion method to effectively integrate the proposed hard and soft traits.
- Analyzing the proposed biometric traits to explore their interactions and correlations between the front and side viewpoints and to identify the most effective and highest-performing ones, along with evaluating and comparing the performance of the proposed approach in augmenting zero-shot side face recognition.

This paper is organized as follows: Section 2 overviews the literature review of recent advancements in face recognition, including the approaches of traditional biometrics, soft biometrics, and multi-modal biometric fusion. Section 3 presents the suggested biometric system framework and research methodology. The results and discussions are given in Section 4. Finally, the paper’s conclusion and suggestions for future work are provided in Section 5.

## 2. Related Works

This section covers research articles pertinent to our work and gives a brief overview of the most recent advancements in face recognition approaches.

### 2.1. Traditional Face Recognition Approaches

Most of the face recognition research mostly adopted traditional approaches, which used traditional techniques for hard feature extraction and biometric template matching, employing a variety of machine learning and deep learning algorithms. In deep learning, the process of regaining a facial geometry from a face image is known as face reconstruction and is used as a model input. In [10], they introduced a face detection and recognition model using the face mesh approach to train a deep neural network. Their model used a set of real-time images in addition to 1700 images from the LFW dataset to detect faces by the Viola-Jones face detector, followed by 468 face landmarks with X, Y, and Z coordinates that represented the face mesh. The Blaze face model located and recognized these landmarks, which label the surrounding area to reconstruct the complete face. The BU3DFE dataset was used to assess the accuracy of face reconstruction from different perspectives. The model achieved 94.23% accuracy under various constraints.

The focus of some approaches was on the challenges of multi-pose faces. Instead of reconstructing faces, other approaches relied on deep learning generative adversarial networks (GANs) to generate new facial samples. Profile-to-frontal coupled GAN (PF-cpGAN) was introduced as a novel method for profile-to-frontal face recognition [11]. This approach was evaluated using several databases, including CFP, CMU Multi-PIE, IJB-A, and IJB-C. A notable progress had been made in real-world face identification with increased front view accuracy (up to 97.7% on IJB-A side frontal variant angle α = 30°) and the accuracy of very slight changes in view in other scenarios (85.3% at ±5° on the driving KITTI dataset). For enhanced face identification of front-to-profile faces, a suited approach was proposed based on the deep residual equivariant mapping (DREAM) block to bridge the gap between frontal and profile face representations [12]. According to experimental findings, it attained a good performance in profile face recognition on various datasets, including CFP and IJB-A, as well as based on other architectures like ResNet-18 and ResNet-50.

Many approaches focused exclusively on side face recognition using other vision-based techniques. They experimented with diverse geometric and texture-based methods for training and testing on side-face images. The authors of [13] proposed a side-view face recognition system for home safety applications, using a new video face database UT-DOOR and cross-database CMU Multi-PIE. The system used multiple feature extraction techniques like principal component analysis (PCA), linear discriminant analysis (LDA), local binary pattern (LBP), and histogram of oriented gradients (HOG) to detect facial landmarks accurately for identification. They achieved accuracies of 92.7% and 96.7% on the CMU Multi-PIE and UT-DOOR databases, respectively. With more focus on the side-face scenario, the study in [14] introduced a perimeter curve-based face identification technique for profiles and facial silhouettes using the GTAV face database. It involved skin color segmentation, curve profiling, AdaBoost ear detection, and modified Hausdorff distance matching. Its reported results showed a false rejection rate of 3.17% in the verification tasks. However, it had limitations due to reduced efficacy on images with fewer facial features.

In [15], they focused only on the front face and aimed to develop an automatic facial recognition system for visually challenged individuals. A collection of human images with faces was generated for training, and a model was created to detect and distinguish between known and unknown faces and eventually produce audio output. The Haar cascade classifier and local binary pattern histograms (LBPH) techniques were used for face detection, and the Euclidean distance algorithm was used to compare detected faces to the trained dataset. The system achieved an accuracy of approximately 93%.

### 2.2. Face Recognition Using Soft Biometrics

The soft biometrics field has been integrated into many recognition systems to contribute to enhanced human identification. Some research studies focused on face recognition and identification using soft biometric traits. A recent study highlighted the importance of profile views in facial recognition and suggested using side-to-side faces as a biometric matching method for training and testing [16]. They proposed a technique for recognizing faces by evaluating their facial profile features using comparative soft biometrics. They used a ranking system for 33 features from the XM2VTSDB dataset and added seven more traits associated with a face profile. They used the Appen crowdsourcing platform for labeling, ensuring high-quality annotations. Mutual information (MI) and Pearson’s correlation coefficient were used to measure the relationship between attributes, where their method achieved a 96% recognition rate by *k* nearest neighbor (*k*NN).

Similarly, the study in [17] explored face recognition algorithms in video surveillance systems, focusing on the challenges of front-to-side face view. The authors tested their proposed algorithm, which used color and texture-based attributes from patches of skin, clothes, and hair for front-to-side face recognition. They used 265 subjects from the FERET color dataset and used soft biometric features, skin color, and shirt color. The system retrieved patches automatically based on facial and eye coordinates, with specific guidelines for each trait. Though the technique showed viability for video surveillance applications, robust real-life re-identification capabilities still require more development and further improvements.

A later research exploration aimed to understand user navigation in a database of front-face images [18]. It evaluated facial aspects for identification, compared machine and human interpretations of facial features, and proposed a new technique for obtaining and authenticating faces based on relative features. Soft biometric variables like facial features and demographic data were used to identify individuals in surveillance databases. Experiments were performed using FERET and LFW datasets, focusing on facial and global categories. Deformable models like active shape, active appearance, and constrained local were used to extract visual information from face images. Their results showed that comparative features can significantly increase the identification accuracy and search efficiency in large datasets. Another study suggested enhancing a primary front-face identification system using soft biometric features like facial dimensions and skin and hair colors [4]. The study used the Faces94 dataset for experiments, where the combination of soft biometrics increased the recognition rate from 91.85% to 95.29% and improved the equal error rate from 3.37% to 1.83%.

A different study highlighted the importance of using soft biometric features, such as gender, ethnicity, and facial marks, to improve face recognition accuracy, especially in challenging scenarios like occlusion and identical twins [19]. An automatic facial mark detection tool was developed using the Active Appearance Model, Laplacian-of-Gaussian blobs, and morphology operators. The algorithm’s rank-1 accuracy increased from 92.02% to 93.90%, enabling face identification from obscured videos and distinguishing between identical twins.

### 2.3. Biometric Feature-Level Fusion for Face Recognition

Combining various techniques is a common practice in related literature. In this approach, a research study focused on combining different traditional feature extraction algorithms to augment face identification [20]. They explored three commonly used descriptors for identifying multi-view face features and proposed a method for improving data representation using feature-level fusion. However, this might result in high dimensionality, potentially affecting classification performance. Instead, they used canonical correlation analysis (CCA) to combine various feature sources for facial image classification, including LBP, HOG, and global image structure tensor (GIST) descriptors. Applying this method to four datasets, AR, CVL, Georgia Tech, and MUCT, they found that combining LBP and GIST features and using CCA significantly enhanced the identification rate in face recognition tests.

A feature-level front-face recognition system was proposed using multi-resolution singular value decomposition (MSVD) fusion, PCA for global features, LBP for local features, and MSVD again for concatenated features [21]. A multi-layer perceptron artificial neural network aggregated and classified feature vectors using 10,000 images from the ORL dataset. The system demonstrated high recognition accuracies ranging from 96.19% to 97.78%. Another study also used the ORL and FRAV2D databases together with camera-captured images. That study aimed to enhance front-face recognition accuracy by integrating multiple feature extraction methods through data fusion techniques [22]. They used two fusion methods: feature-level fusion, which combined feature vectors extracted using PCA, LBP, and discrete cosine transform (DCT), and decision-level fusion, which applied feature extraction methods independently and then merged their results via majority vote. They used Manhattan, Euclidean, and cosine distances in the classification training and testing phases. Despite promising results in current face recognition investigations, further research and extended analyses are needed to determine the practical and superior fusion strategy.

A novel multi-modal biometric system was introduced to enhance identification accuracy by combining facial and fingerprint features [23]. In the suggested multi-modal approach, the feature dimensionality is reduced by PCA and LBP feature extraction from face and fingerprint images. In order to provide a single representation for biometric identification, the collected features were subsequently integrated using the weighted summation fusion technique. After collecting features from fingerprint and facial data, these distinct traits were joined to form a composite feature vector. The suggested method maintained a low computing cost while demonstrating a 97.5% recognition rate inferred by the probabilistic neural network (PNN) used as a classifier.

Despite the recent advancement of the face recognition research field, the challenges of side face views remain inadequately addressed, gaining little research interest and concentration. The utilization of the soft biometrics and semantic features that can be observed from front to side and the fusion with the capabilities of vision-based techniques like pre-trained deep learning models have yet to be extensively investigated, which thus can narrow the gap for such a challenging scenario and enhance face recognition performance.

## 3. Proposed Methodology

**Figure 2:**
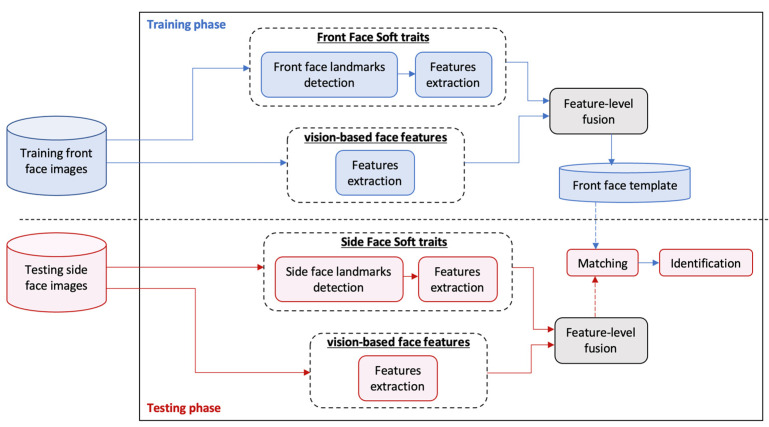
Overview of our proposed system framework.

Our system framework has been developed to use the front-face images only in the training phase. After that, the side-face images, as challenging unseen data, are solely used for testing. In other words, we adopt a challenging real-life scenario that necessitates using side-face images as the only available query information to test a system that was trained only on front-face images and never exposed to any side-face image samples. Figure 2 depicts the framework, which consists of four main modules: hard (traditional vision-based) face feature extraction, face soft trait extraction, feature-level fusion, and biometric-template matching for identification. This system uses the CMU Multi-PIE (Multi Pose, Illumination, Expressions) dataset. The vision-based part is implemented to apply deep learning pre-trained models ResNet-50 and VGG-16 for hard deep feature extraction, where this module is conducted similarly for training and testing. In the face soft trait extraction module, distinct multi-landmark detection techniques are employed to locate a group of facial key points during the training and testing phases. Then, face soft traits are automatically extracted and labeled after detecting and calculating the distances between the key points of interest. After analyzing, selecting, and adopting the most viable subset among several potentially effective facial soft traits, the feature-level fusion is applied to the hard and soft traits derived in the previous stages. In the last stage, a support vector machine (SVM)-based classifier is used to match biometric templates for identification. Noteworthy, in this research methodology, the mapping of the inferred face biometric information from the front-to-side viewpoint is thoroughly investigated and extensively analyzed.

### 3.1. Face Image Dataset

In our proposed system, we need a dataset that offers both frontal and full-side face view images per person. Therefore, we use a collection of facial images called the CMU Multi-PIE (Multi Pose, Illumination, Expressions) Face Dataset, as it is suited to attain the objective of this research study. This dataset consists of 337 subjects’ faces captured in various poses, lighting conditions, and expressions [24]. Fifteen distinct views constitute the pose range, which captures a face from side to side. Nineteen spotlights positioned around the room were used to simulate changes in illumination. Additionally, it has female and male subjects of diverse ages, ethnicities, and expressions. We use an available preprocessed version of the dataset, where all faces are detected and stored as images in RGB colors, PNG format, and 128 × 128 resolution [25]. For our system, we select 50 subjects of different genders, ages, and ethnicities.

**Figure 3:**
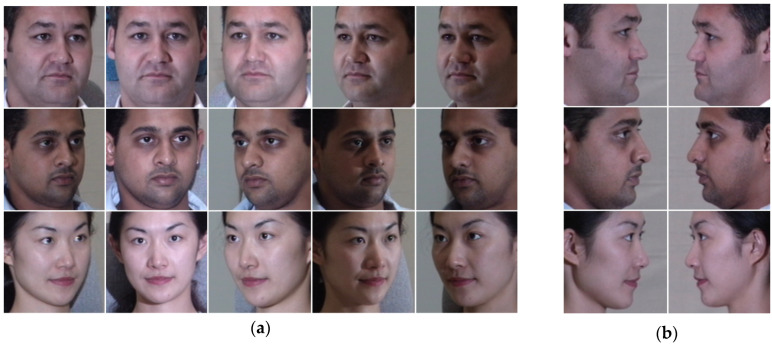
Samples from the CMU Multi-PIE dataset, where (**a**) front face samples of training images of different angles; (**b**) samples of testing images represent right side α = +90° and left side α = −90°.

In this research, since we are interested in face anthropometry, we describe the *front-face* as the face that we can see and detect all its components (mouth, nose, two eyes, and two eyebrows) regardless of its pose angle, whereas the *side-face* shows only the half face components (side-mouth, side-nose, one eye, and one eyebrow). As such, in the training phase, we choose 16 images per subject under multi-pose angles and multi-illumination without expression. Additionally, in the testing phase, we choose two images per person (one for the right side α = +90° and the other for the left side α = −90°). The total number of selected data is 800 images for training and 100 for testing, while each training image undergoes further analysis by an automatic front landmarks detector to assess the degree of pose and decide whether to accept it for training if it is compliant with the front-face constraints, ensuring the exclusion of any side-face or side-face-like images from training data. Figure 3 provides some image samples from the used dataset.

### 3.2. Face Soft Biometric Traits

#### 3.2.1. Face Landmarks Detection

**Figure 4:**
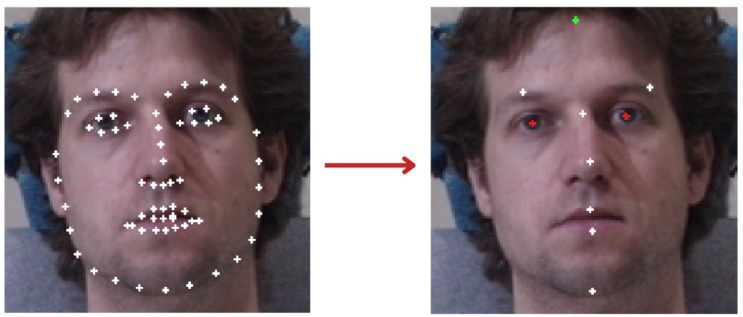
Selecting the subsect key points of interest from the automatic Dlib 68 key points, comprising: (8: chin), (27: nasion), (30: nose tip), (51: upper lip), (57: lower lip), (19: left eyebrow), (25: right eyebrow), in addition to further estimated automatic key points for forehead and eye pupils.

Before extracting the soft biometric features, the desired facial landmarks need to be first detected. Face landmark detection is a fundamental technique in computer vision that refers to the process of locating significant facial features and adding key points to them [26]. These important key points, referred to as landmarks, include many facial features such as facial contours, lip borders, nose tips, corners of the eyes, etc. By marking these landmarks, a wide range of eventual tasks can be fulfilled, including facial recognition, emotion recognition, facial analysis, and augmented reality applications. In this research, for the training phase, we use the Dlib library’s pre-trained facial landmark detector to locate 68 (x, y)-coordinates corresponding to facial features on the front face [27]. This library is primarily developed to detect the front-face components in multiple poses. After applying it to the 800 training images, the Dlib successfully accepts 738 images as front faces and rejects the others to be excluded from the training subset, as summarized in the data distribution shown in Table 1. According to our proposed soft traits, we need a specific landmarks list along with additional new key points for the forehead and eye pupils automatically detected as shown in Figure 4, where the added new forehead and pupil key points are highlighted in green and red, respectively.

**Figure 5:**
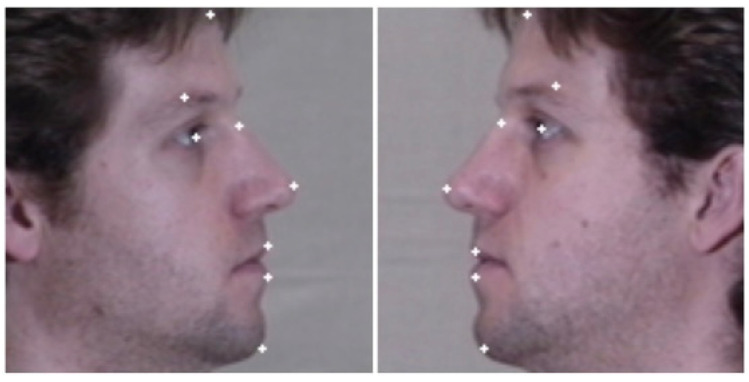
Manual side face key points annotation for right and left face sides.

For the testing phase, to the best of our knowledge, no available ready-made and ideally applicable models to our need for detecting side face landmarks. Therefore, based on the same location of Dlib front landmarks, we apply further manual key points annotation to the side faces. Figure 5 shows the applied manual side-face annotation, which is a common technique in computer vision and machine learning fields to provide labeled datasets for validation and investigation purposes. We utilize the RectLabel tool since it provides an easy-to-use interface that enables the annotation of images. It provides the capabilities for managing, organizing, and altering annotations in addition to supporting several picture formats [28]. The labels are saved in extensible markup language (XML) file format, each file contains the (x, y)-coordinates of the side face landmarks. The tool also facilitates the usage of labeled data in model training pipelines by integrating with popular machine learning frameworks and being compatible with macOS, as per the machine used in the current experimental work.

#### 3.2.2. Face Soft Traits

We propose a new set of soft features based on face anthropometry to augment both side and front face identification. Anthropometry is defined as the measuring of the human body and its constituent parts [29]. Facial anthropometry is a subfield of anthropometry that is particularly concerned with the quantitative analysis of facial feature measures and proportions. This field of study requires measurements of several facial features, including the length of the jawline, the breadth of the eyes, and the space between the nose and the contour. These measurements can be essential in medical sectors for plastic surgery, diagnostic and treatment planning, forensic science, and studies for distinguishing between ethnicities [30]. Facial anthropometry is relevant to the biometrics domain, especially biometric facial recognition technologies, which use a person’s distinct facial measurements to identify and authenticate them.

**Figure 6:**
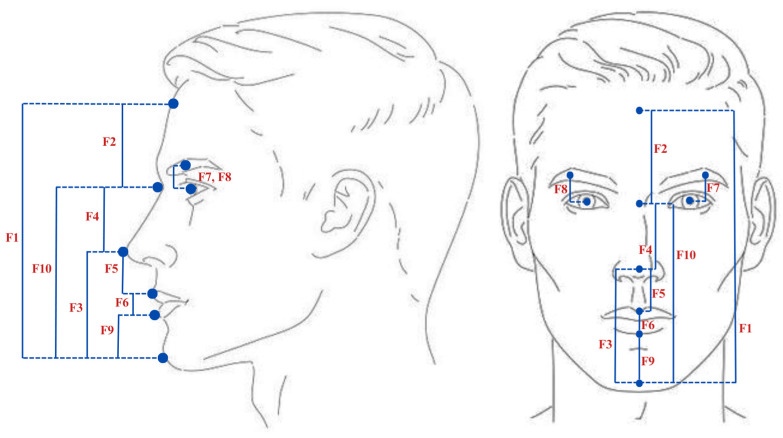
The positions and visual representations of 10 proposed distance-based face soft traits.

Here, we propose 33 anthropometric-based soft face traits that are easily understood and automatically extracted, divided into 10 facial distances and 23 facial ratios. To bridge the front and side face views, we focus on the vertical distance traits, which can be invariant and immune to changes from front to side as shown in Table 2. Moreover, the positions of the 10 facial distances (features F1–F10) are illustrated in Figure 6. After extracting these 10 distances, we take the relative proportions between them to derive 23 new ratios. In anthropometry, the facial measurements are directly measured in millimeters or centimeters [31,32]. In our system, each distance trait is first detected by the facial landmarks approach [33]. Then, for each trait, we calculate the Euclidean distance between the facial key points to determine the most appropriate corresponding (relative) semantic label automatically [34]. All 33 traits are semantically labeled using a bipolar five-point scale [very large—large—medium—small—very small] as ordinal attributes. In order to assign applicable labels for distance traits, we take into consideration the following aspects: calculating the mean Euclidean distance for each trait and then comparing each distance value within the same trait to its corresponding mean distance. After that comparison, we assign the semantic labels in relative form as follows: “very large” (the value is larger than the mean, within a range larger than +5 units per Euclidean distance), “large” (the value is larger than the mean, within a range between +2 and +5), “medium” (the value is equal or close to the mean, within a range of ± 2), “small” (the value is smaller than the mean, within a range between −2 and −5), “very small” (the value is smaller than the mean, within a range smaller than −5). After extracting distance values, we infer the proportions for the 23 ratio traits (features F11–F33), as elaborated in Table 3. These facial ratio traits are fractional numbers; thus, we use the percentile to determine the labels to be assigned [35]. The percentile formula is:

(1) $$P_{k}=\left(\frac{k}{100}\right)\times \left(N+1\right)$$

where $P_{k}\;$ represents the percentile value, k denotes the target percentile, such as 10 for the $10^{th}$ percentile, and N is the total number of all the data points. For each ratio trait, we calculate the $10^{th}$, $30^{th}$, $70^{th}$, and $90^{th}$ percentiles. Then, each ratio value is compared to all percentiles to assign a suited label, such that: “very large” (the ratio value is larger than the $90^{th}$ percentile), “large” (the value is within a range between $70^{th}$ and $90^{th}$ percentiles), “medium” (the value is within a range $30^{th}$ and $70^{th}$ percentiles), “small” (the value is within a range $10^{th}$ and $30^{th}$ percentiles), “very small” (the value is less than the $10^{th}$ percentile). Besides our proposed soft traits, we also employ the global soft biometrics traits that can be readily deuced as supplementary features, such as an individual’s gender and ethnicity [36]. We include a limited set of six global soft biometrics that are frequently and effectively employed earlier to examine the efficacy and reliability of enhancing the traditional face recognition technique [37].

Table 4 represents the six used global soft traits and their corresponding semantic labels in (categorical) absolute form.

#### 3.2.3. Analysis of Face Soft Traits

We use various methods in this study to examine, investigate, and analyze the proposed face soft traits. Utilizing different analysis methods helps obtain an extensive understanding of their capabilities and explore how well they improve recognition performance.

- Analysis of variance (ANOVA):

In addition to providing a coherent description of the human face, each suggested soft trait can offer discriminative information from different perspectives to improve subject-to-subject differentiation within the database. Therefore, in order to evaluate the statistical significance of each soft trait, a one-way analysis of variance (ANOVA) is utilized [38]. Evaluating the variances between and within groups assists us in determining how and which traits contribute the most to recognition. The F-ratio statistic serves as a significance score to rank the face soft traits by performance, and it is calculated as follows:

(2) $$F-ratio=\frac{Variance\;between\;groups}{Variance\;within\;groups}=\frac{\sum_{i}n_{i}{\left({\bar{X}}_{i}-\bar{X}\right)}^{2}/\left(k-1\right)}{\sum_{ij}{\left(X_{ij}-\bar{X}\right)}^{2}/\left(N-k\right)}\;$$

where N is the total number of observations obtained for all groups, k is the quantity of distinct groups (persons) in the dataset, $n_{i}$ is the total observations of the group i, ${\bar{X}}_{i}$ represents the mean observation of the group i, $\bar{X}$ represents the mean of all the N observations, $X_{ij}$ represents the $j^{th}\;$ observation in group i. Namely, the larger the inter-class variance than the intra-class variance, the higher the F-ratio value is. Hence, this specific trait is deemed a more significant and effective measure of identification [39], where a higher F-ratio denotes a more important soft trait to identify and a more significant soft trait to identify. Remarkably, it is apparent that each of the 33 traits contributes to providing unique information for identification. Table 5 reports the deduced F-ratio and *p*-value per face soft trait with a significance level of *p* < 0.01.

- Pearson’s *r* correlation:

The invariant viewpoint from the front-to-side face is essential. In this paper, since we are considering that the front and side face views can be interrelated by some invariant features, we examine the 33 proposed face soft traits to understand and determine to what extent they are invariant from the front-to-side face view. Analyzing the correlations between labels that describe the same facial traits from the front and side perspectives can help determine which features are more robust, dependable, and resilient to changes in viewpoint [40]. We utilize the Pearson correlation coefficient, which measures the linear relationship between variables [41]. A sorted list of Pearson’s *r* correlations that are computed between front-view and side-view labels of the same face trait is shown in Table 6. Higher positive correlation values, near or equal to 1, indicate that those traits are more invariant and consistent across the front and side viewpoints.

**Figure 7:**
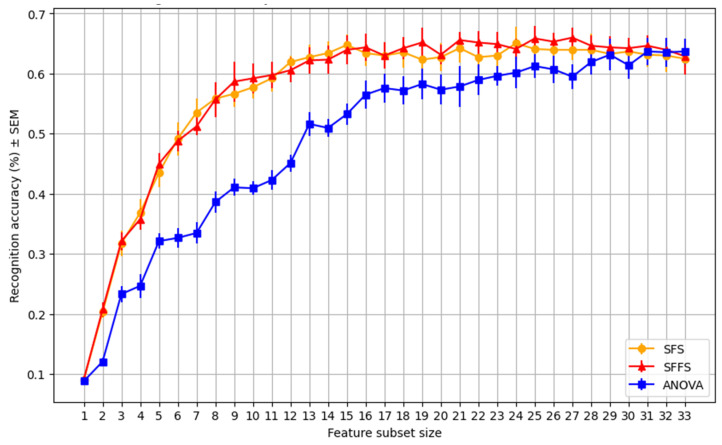
SEM value growth trend during feature subset selection using three different methods, showing how each chosen feature incrementally contributes to recognition improvement.

- Feature subset selection:

This process aims to analyze and investigate the feasibility of enforcing an effective feature subset selection method on the proposed face soft traits. This way, a feature subset can be nominated, comprising the most substantial features that contribute most to the recognition task and potentially achieve increased performance with minimal features. We use sequential forward selection (SFS) and sequential floating forward selection (SFFS) to evaluate the feature subsets in recognition [42,43]. SFS moves forward by only pursuing the best-performing feature among the remaining features. As a result, the chosen subset size increases as long as adding any new feature from the yet unselected ones improves performance. The forward SFS and sequential backward selection (SBS) are combined in the SFFS algorithm. By adding the retracing step in the backward direction, SBS improves upon SFS in terms of search performance. This stage of backtracking aids in removing a trait that reduces learning effectiveness. The enhancement is performed during the selection process, making it a conditional phase. Figure 7 shows the significance assessment of selecting features during the recognition task in isolation (as the only used features). The feature subset selection procedure shows a trend of growth in structural equation modeling (SEM) scores, as pertinent features are added one at a time. The SEM graphical representation illustrates how feature addition affects the model’s overall performance [44]. The resulting curves show that employing a selection of 15–20 soft traits yields the best SFS performance. Additionally, SFFS reaches its highest level in the 19–21 subset and remains steady when more traits are added afterward. Furthermore, the ANOVA ordering by F-ratio, as feature subset selection, offers a curve that indicates slower growth and better performance when all 33 soft traits are used. Note that further extended experiments are conducted, as in Section 4, to determine the effectiveness of these selections when combined with the hard features.

#### 3.2.4. Normalization of Soft Traits

Normalization seeks to balance the scale of each data point, thereby providing a comparable significance to each feature [45]. Feature normalization is mainly necessary to remove the impact of many quantitative features that are measured on various scales, which makes it an essential procedure in feature-level fusion. In statistics and machine learning, z-score normalization (ZN)—also referred to as standardization—is an essential data preprocessing method [46,47]. It ensures that all features are on the same scale by transforming data into a standard normal distribution. This procedure helps prevent the dominance of particular features over others due to scale disparities, which can seriously affect how well machine learning models work. The formula of ZN is:

(3) $$Z=\frac{X-\mu }{\sigma }$$

where Z stands for z-score, the value of the data point for which the z-score is being calculated is denoted by X, the data points’ mean is μ, and σ stands for the data points’ standard deviation.

### 3.3. Vision-Based Face Traits Extraction

Among a wide variety of practical vision-based feature extraction techniques for face recognition, deep learning models are well-researched methodologies that have repeatedly demonstrated their power and ability to serve as highly discriminative feature extractors for a variety of recognition applications. With the fast-expanding field of deep learning, pre-trained models have become an essential building block for many computer vision applications [48]. To narrow and resolve the gap between frontal and side face view, we use the pre-trained models because they are designed to capture a wide range of facial features and are robust to changes in position, lighting, and expressions. Moreover, the models trained on various datasets can perform better when generalizing to new unseen data with limited training datasets. Therefore, in this research, the standard hard biometric features for experimental work have been extracted using VGG−16 and ResNet-50 pre-trained deep learning models, described in [49,50], to be used alone and then fused with the proposed soft traits for identification performance evaluation and comparison.

- VGG-16:

Visual geometry group (VGG) is a deep convolutional neural network (CNN). The term “deep” in this context specifically refers to VGG-16’s sixteen convolutional layers [51]. It is presently considered one of the most potent image recognition architectures. The ImageNet database was used as the training set for the VGG-16 network. Due to its comprehensive training, the VGG-16 model provides exceptional accuracy even with small image datasets for fine-tuning. It standardly preprocesses the images to be resized to 224 × 224 resolution and RGB colors. The architecture of the VGG-16 network contains a 3 × 3 tiny receptive field and 13 convolution layers grouped into five blocks, each ending with a max pooling layer of size 2 × 2. After the final max pooling layer, there are three fully connected layers. As the last layer, it employs the SoftMax classifier, and for every hidden layer, the rectified linear unit (ReLU) activation is used [52].

- ResNet-50:

The residual network (ResNet) was introduced as a novel, versatile neural network [53]. It has multiple versions that function according to different principles, yet they all have different numbers of layers. ResNet-50 model can accommodate 50 layers of neural networks, and it has more than 25.6 million parameters. It consists of a fully connected layer, convolution, and an identity block (input = output). The input value of the initial block or signal is matched by the identity x. Hence, the output value of the residual block is the total of the block’s input value and the output values of its internal layers [54].

### 3.4. Bioemric Trait Fusion

**Figure 8:**
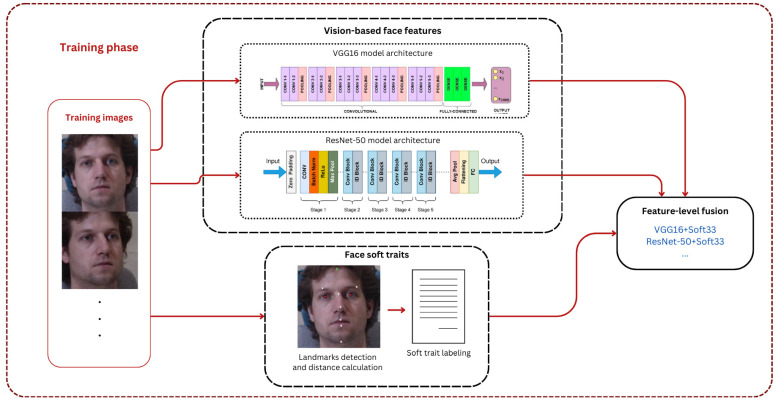
Extracting and fusing different vision-based deep features at the training phase.

In biometric systems, fusion approaches are frequently divided into four levels: decision, score, rank, and feature [55]. These approaches can improve the accuracy, robustness, and dependability of the system as a whole by combining various biometric inputs. Such fusions could provide a more reliable and thorough identification by combining data from several biometric process stages [56]. In our system, feature-level fusion is implemented to combine the traditional/hard and the proposed semantic/soft features. We synthesize a single feature vector through the combination of discriminative information from several feature sources since it is simple and frequently used for augmenting performance. Figure 8 is a partial focus on the proposed framework that displays the detailed feature extraction and fusion processes during the training phase.

### 3.5. SVM-Based Classification

In order to accomplish the face recognition objective of the suggested framework, we employ an SVM-based classifier to examine the efficacy and validity of the fusion of soft and hard face biometrics [57]. SVM as a binary linear classification method, divides the classes based on the greatest distance (optimal margin) between the borderline instances (support vectors). Data is segregated using a linear surface (hyperplane) in a new high-dimensional feature space, created by mathematical functions called kernels [58]. The linear kernel formulation can be provided by:

(4) $$f\left(x\right)=w\cdot x+b$$

The input feature vector is denoted by x, the weight vector w is learned during training, and the bias is indicated by b. We concentrate on the classification using a supervised linear support vector classifier (SVC) as it can be robust against overfitting and efficiently handle high-dimensional data, which is beneficial for numerous applications [59].

## 4. Experiments

In this section, we discuss the evaluation metrics applied in our study and provide a thorough analysis of the experimental results. The performance analysis aims to determine the efficiency of the proposed face soft traits in enhancing and augmenting the performance of zero-shot side face recognition. All experiments are conducted on Google Colab, utilizing CPU-based processing and PYTHON3.

### 4.1. Evaluation Metrics

Several metrics are used to evaluate and compare the viability and performance of the proposed approach against traditional counterparts in augmenting zero-shot side face recognition. The confusion matrix is a conformed and frequently utilized instrument for practical assessment in this research context. It is typically carried out to compute four statistical distributions: true positive (TP), true negative (TN), false positive (FP), and false negative (FN), where each conveys a distinct pairing of the projected and actual classes [60]. The confusion matrix can further be used to infer additional informative performance indicators, including the following:

- *Accuracy*: the proportion of correctly classified instances among all instances.

(5) $$Accuracy=\frac{TP+TN}{TP+TN+FP+FN}$$

- *Precision*: the accuracy of positive forecasts (positive predictive value).

(6) $$Precision=\frac{TP}{TP+FP}$$

- Cumulative match characteristic (CMC): a metric employed to assess the effectiveness of identification systems (as one-to-many). It evaluates such systems based on their ability to rank by identification match scores. It yields a ranked list of registered candidates from the dataset based on the relative ranking of matching scores for each biometric sample [61].
- Receiver operating characteristic (ROC): represents the performance graphically and gives the possibility to view how the trade-off between the true positive rate (TPR) and false positive rate (FPR) changes along gradually varying categorization levels [62].

(7) $$\mathrm{ROC}=\frac{P(x|positive)}{P(x|negative)}$$

- Equal error rate (EER): represents a level of equality between the false rejection rate (FRR) and the false acceptance rate (FAR). It is the level at which the likelihood of the system incorrectly accepting a nonmatching person is equal to the likelihood of incorrectly rejecting a matching person. In biometric security systems, EER is an essential measure, since it maintains a balance between accessibility and security.

(8) $$\mathrm{FRR}=\frac{FN}{FN+TP}$$

(9) $$\mathrm{FAR}=\frac{FP}{FP+TN}$$

(10) $$\mathrm{EER}=\frac{\mathrm{FRR}+\mathrm{FAR}}{2}$$

- Area under the curve (AUC): the ability of a model to differentiate between two classes. Better performance is indicated by higher numbers, which range from 0 to 1.

### 4.2. Experiments and Results

We utilize the CMU Multi-PIE dataset, which is publicly available for research purposes, for all our experiments. At first, we used the SVM classifier to examine the performance of the two traditional/hard pre-trained models (ResNet-50 and VGG-16) separately. The initial accuracy results for VGG-16 and ResNet-50 were both 62%. After that, to determine the most optimal fusion outcome, we conducted two feature-level fusion scenarios and compared the fusion outcomes with the initial outcomes.

#### 4.2.1. Hard and Soft Biometric Trait Fusion

To verify the viability of the proposed soft traits, we performed the first augmentation approach by fusing the 33 soft traits with each feature vector of the ResNet-50 and VGG-16 pre-trained models. Furthermore, the performance of integrating all three VGG-16, ResNet-50, and soft traits was subsequently assessed and compared with the other counterparts. The features extracted from ResNet-50 are 2048, while the VGG-16 features are 25,088, which is higher-dimensional. For this reason, we applied a PCA-based reduction to the nascent VGG-16 features. We calculated the lowest number of principal components required to preserve 0.95 percent of the variation in the preliminary features. Moreover, because far more hard-face features are retrieved and employed per fused vector than soft-face features, traditional biometrics could prevail in matching decisions and reduce the usefulness of soft-face biometric information. Therefore, we allocated the soft features to effective weight coefficients through empirical experimentation. The optimal coefficient to obtain 68% is 5.5 when combining the soft traits with the VGG-16 features. Additionally, this coefficient keeps providing the same accuracy of 68% when combining all three techniques: soft, ResNet-50, and VGG-16 features. Nonetheless, we obtained a higher performance result of 72% when combining the soft traits with the ResNet-50 without using the weight coefficient factor since it did not attain any performance enhancement to this combination.

We repeated the same experiments, but we applied a feature subset selection method to the proposed 33 soft traits by SFS, SFFS, and ANOVA selections to determine the best features for the performance. The experimental results reveal that the feature subset selection by these three methods could not achieve higher or similar performance when fused with face hard features, and this emphasizes that all 33 soft traits are significant and influential in augmenting zero-shot side face identification to achieve the highest possible performance.

#### 4.2.2. Hard, Soft, Global-Soft Biometric Trait Fusion

For further augmented identification, we integrated six global soft traits with all three combinations described in the above section. The VGG-16 achieved 68% accuracy when combined with the 33 soft traits and the 6 global traits, weighted with a 5.5 coefficient, yet it did not demonstrate any discernible improvement across all combinations in which VGG-16 was involved. The highest performance enhancement was observed when combining ResNet-50 with the 33 soft traits and the 6 global traits, with an optimal soft train weight coefficient equal to 3.5, where identification performance was significantly augmented, and the accuracy increased to 85% and precision to 81.13%. Consequently, we improved the accuracy of the baseline traditional VGG-16 and ResNet-50 techniques by 6% and 13%, respectively. All experimental findings for various soft-based fusion versus traditional features are reported in Table 7.

These findings demonstrate that the proposed soft face biometrics’ capabilities may be subsequently applied to extended fusion and improved performance, which can outperform traditional features when used alone. It ought to be noted that the SVM classifier improved all recognition rates. Particularly, VGG-16 and ResNet-50 had an accuracy of 62% before combining them with the soft face features. The accuracy increased between 68% and 85%, signifying the potential of fusion techniques to produce promising zero-shot side face recognition rates.

**Figure 9:**
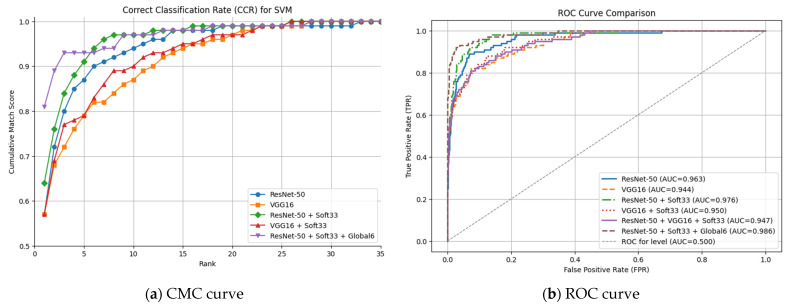
Zero-shot side face (**a**) identification and (**b**) verification performance evaluation and comparison between hard-based traditional and newly proposed soft-based augmented approaches.

Accordingly, it is apparent in Figure 9 that soft face biometrics exhibit a notable enhancement in efficiency in identification and verification, as shown by the analysis of ROC and CMC, respectively. It is noticeable that in rank-35 the majority of models attain cumulative match scores of almost 100%. Based on the CMC curves, the correct classification rate (CCR) is the top match score (rank = 1), while the CMC average sum scores can be computed for any top *k* ranks, such as 5 and 10. Particularly, at rank-1, the model ResNet-50_soft33_global6 obtained the highest score and continued to achieve higher scores than the other approaches predominately. Despite the addition of soft traits, the VGG-16 model is obviously less effective in augmenting performance than the other models, as proved by its CCR scores. The detailed identification and verification performance is reported in Table 8, deduced from the CMC analysis and ROC curve represented by FRR against FAR for different threshold values (expressed by AUC and ERR). Although all three soft-based augmented approaches outperform the other two traditional nonaugmented approaches, ResNet-50_soft33_global6 receives the highest performance by all means, including the superior 97% average score of the top ten matches, best AUC of 98.6%, and least EER of only 0.062.

Eventually, Table 9 compares our research on soft biometric-based augmented zero-shot side face recognition with three related studies from the literature (reviewed in Section 2.1 and Section 2.2), highlighting the effectiveness of our approach when fusing the traditional and the proposed soft biometrics. The first study used hard biometric techniques only and trained and tested the system on the same side face view. Similarly, for the second, except that they used just soft biometric features. As for the third, they used front-to-side face re-identification by three patches for three soft traits. On the other hand, our system was trained using only frontal faces of many angles and tested using unseen images of side faces (left side, right side) on a zero-shot basis while fusing the hard deep features with the face-soft biometrics. It should be recalled that since the experimental circumstances and databases differ, some potential comparison elements are not applicable.

## 5. Conclusions

This study presents a set of new soft face biometric traits derived from the human anthropometric face measurements, particularly those consistently and invariantly observed from a front-to-side face view. It then explores the possibility of integrating these soft traits to enhance traditional (hard) facial biometric features extracted using the deep learning pre-trained models VGG-16 and ResNet-50. All experiments were conducted on the CMU Multi-PIE dataset by selecting 50 subjects. The total training images were 738, comprising different frontal images per subject, whereas 100 of both (left-side, right-side) images per subject were used for testing. To investigate the significance of the proposed soft face traits, ANOVA was utilized, highlighting the potency of each soft face trait for enhancing identification and verification performance and increasing the accuracy of traditional face features. Furthermore, to understand and bridge the semantic gap between the same soft face traits inferred from the front and side views, we applied Pearson’s r correlation to assess their consistency and demonstrate how likely each soft trait is to be observed invariantly from both viewpoints.

As a baseline for testing and comparison, traditional facial recognition approaches yielded 62% accuracy for both VGG-16 and ResNet-50 via SVM. These accuracies were raised to 68% and 72%, leveraging feature-level fusion with the soft biometrics in their augmented versions. Additionally, by fusing the ResNet-50 with the proposed 33 face soft traits and the six global soft traits, we achieved the highest accuracy of 85%. In order to improve fusion and recognition accuracy, distinct weight coefficients have been assigned to the soft face traits when paired with traditional features. We noted that applying feature subsets selection by SFS and SFFS on the proposed face soft traits did not improve or maintain performance when fused with the traditional features. Thus, we concluded that all 33 soft traits are effective and significant for better-augmented performance. The proposed approach highlighted how the fusion of hard and soft facial traits surpasses the hard features individually. We demonstrated that the proposed soft face biometric traits could augment the hard face features and contribute to overcoming the semantic barrier between humans and machines to augment the side recognition ability. Consequently, our system proved its ability to recognize both left-side and right-side face images in zero-shot scenarios after being trained only on front-face images.

The primary limitation of this study is the landmark detection of the side-face view. To the best of our knowledge, no reliable, automatic, ready-made, and applicable models exist for robustly detecting side face landmarks of our interest. In contrast, many automatic techniques are available for the front-face view. Fortunately, based on existing literature, our research investigation is considered among the first research works that substantially use face anthropometric capabilities to automatically extract invariant front-to-side soft face biometrics for effective hard-soft biometric fusion and augmented side face recognition purposes. It is evident from this preliminary research that more functional front-to-side face soft traits could be investigated and derived from human anthropometrics in future studies. Additionally, it might be feasible to devote future efforts to creating reliable detectors to automatically extract side face landmarks or leverage crowdsourcing platforms to enable human annotator labeling. Additionally, we will explore other datasets that provide both front and side face images in different higher/lower resolutions to validate our approach further and compare its performance using different classifiers, enabling performance variation analysis and superior classifier investigation. A potential future direction can be focused on an extended comparative study of using the proposed approach for augmenting deep features extracted by several different specialized facial feature extraction/embedding models. To further improve human recognition systems, this study could additionally set other research avenues for researchers to pursue, leveraging multidisciplinary research domains, such as extended invariant human anthropometry and multi-pose face recognition.

## Figures and Tables

**Table 1:** Distribution of the used subset of the CMU Multi-PIE dataset.

Data Distribution	Training	Testing
Number of images	738	100

**Table 2:** Description of 10 proposed distance-based face soft traits.

ID	Facial Distance Attributes	Description	Semantic Labels	Label Form
F1	Face height $\left(fh\right)$	Distance between the forehead to chin	[very large—large—medium—small—very small]	Relative
F2	Forehead height $\left(foh\right)$	Distance between the forehead and nasion	[very large—large—medium—small—very small]	Relative
F3	Lower face height $\left(lfh\right)$	Distance between the nose tip and chin	[very large—large—medium—small—very small]	Relative
F4	Nasal length $\left(nl\right)$	Distance between nasion and tip of nose	[very large—large—medium—small—very small]	Relative
F5	Philtrum $\left(ph\right)$	Distance between the nasal tip and upper lip	[very large—large—medium—small—very small]	Relative
F6	Mouth height $\left(mh\right)$	Distance from the lower lip to upper lip	[very large—large—medium—small—very small]	Relative
F7	Eye-to-eyebrow-left $\left(yyl\right)$	distance between left eye to left eyebrow	[very large—large—medium—small—very small]	Relative
F8	Eye-to-eyebrow-right $\left(yyr\right)$	distance between the right eye to right eyebrow	[very large—large—medium—small—very small]	Relative
F9	Chin-to-mouth $\left(cm\right)$	Distance between chin to the lower lip	[very large—large—medium—small—very small]	Relative
F10	Nasion-to-chin $\left(nc\right)$	Distance between nasion and chin	[very large—large—medium—small—very small]	Relative

**Table 3:** Description of 23 proposed ratio-based face soft traits.

ID	Facial Ratio Attributes	Calculation Formula	Description	Semantic Labels	Label Form
F11	Forehead to face-height ratio	$\frac{foh}{fh}$	The proportion of forehead height relative to the total face height	[very large—large—medium—small—very small]	Relative
F12	Lower face to face-height ratio	$\frac{lfh}{fh}$	The proportion of lower face height relative to the total face height	[very large—large—medium—small—very small]	Relative
F13	Nasal length to face-height ratio	$\frac{nl}{fh}$	The proportion of nose length relative to the total face height	[very large—large—medium—small—very small]	Relative
F14	Philtrum to face height	$\frac{ph}{fh}$	The proportion of philtrum distance relative to the total face height	[very large—large—medium—small—very small]	Relative
F15	Mouth-height to face-height ratio	$\frac{mh}{fh}$	The proportion of mouth height relative to the total face height	[very large—large—medium—small—very small]	Relative
F16	Nasion-to-chin to face-height ratio	$\frac{nc}{fh}$	The proportion of nasion-to-chin distance relative to the total face height	[very large—large—medium—small—very small]	Relative
F17	Chin-to-mouth to face-height ratio	$\frac{cm}{fh}$	The proportion of chin-to-mouth distance relative to the total face height	[very large—large—medium—small—very small]	Relative
F18	Eye-to-eyebrow-left to face-height ratio	$\frac{yyl}{fh}$	The proportion of eye-to-eyebrows distance relative to the total face height	[very large—large—medium—small—very small]	Relative
F19	Eye-to-eyebrow-right to face-height ratio	$\frac{yyr}{fh}$	The proportion of eye-to-eyebrows distance relative to the total face height	[very large—large—medium—small—very small]	Relative
F20	Nasal length to nasion-to-chin ratio	$\frac{nl}{nc}$	The proportion of nose length relative to the nasion-to-chin distance	[very large—large—medium—small—very small]	Relative
F22	Mouth-height to nasion-to-chin ratio	$\frac{mh}{nc}$	The proportion of mouth height relative to nasion-to-chin distance	[very large—large—medium—small—very small]	Relative
F23	Chin-to-mouth to nasion-to-chin ratio	$\frac{cm}{nc}$	The proportion of chin-to-mouth distance relative to nasion-to-chin distance	[very large—large—medium—small—very small]	Relative
F24	Philtrum to mouth height ratio	$\frac{ph}{mh}$	The proportion of mouth-to-nose distance relative to mouth height	[very large—large—medium—small—very small]	Relative
F25	Philtrum to chin-to-mouth ratio	$\frac{ph}{cm}$	The proportion of mouth-to-nose distance relative to chin-to-mouth distance	[very large—large—medium—small—very small]	Relative
F26	Philtrum to lower face-height ratio	$\frac{ph}{lfh}$	The proportion of mouth-to-nose distance relative to the lower face height	[very large—large—medium—small—very small]	Relative
F27	Mouth height to lower face-height ratio	$\frac{mh}{lfh}$	The proportion of mouth height relative to the lower face height	[very large—large—medium—small—very small]	Relative
F28	Chin-to-mouth to lower face height ratio	$\frac{cm}{lfh}$	The proportion of chin-to-mouth distance relative to the lower face height	[very large—large—medium—small—very small]	Relative
F29	Eye-to-eyebrow-left to forehead height ratio	$\frac{yyl}{foh}$	The proportion of eye-to-eyebrow distance relative to forehead height	[very large—large—medium—small—very small]	Relative
F30	Eye-to-eyebrow-right to forehead-height ratio	$\frac{yyr}{foh}$	The proportion of eye-to-eyebrow distance relative to forehead height	[very large—large—medium—small—very small]	Relative
F31	Philtrum to nasal-length ratio	$\frac{ph}{nl}$	The proportion of mouth-to-nose distance relative to the nasal length	[very large—large—medium—small—very small]	Relative
F32	Mouth height to nasal-length ratio	$\frac{mh}{nl}$	The proportion of mouth height relative to the nasal length	[very large—large—medium—small—very small]	Relative
F33	Chin-to-mouth to mouth height ratio	$\frac{cm}{mh}$	The proportion of chin-to-mouth distance relative to mouth height	[very large—large—medium—small—very small]	Relative

**Table 4:** Description of used global soft biometrics traits.

Soft Trait	Semantic Labels	Label Form
1. Gender	[Male—female]	Absolute
2. Ethnicity	[European—Middle eastern—Far eastern—South Asian—African—Mixed—Other]	Absolute
3. Age group	[Infant—Preadolescence—Adolescence—Young adult—Adult—Middle aged—Senior]	Absolute
4. Skin color	[White—oriental—tanned—brown—black]	Absolute
5. Skin tone	[Fair—light—medium—brown—dark—very dark]	Relative
6. Facial hair	[None—mustache—beard]	Absolute

**Table 5:** ANOVA-derived ordered F-ratios and *p*-values of the proposed face soft traits.

ID	F-Ratio	*p*-Value	ID	F-Ratio	*p*-Value	ID	F-Ratio	*p*-Value
F27	40.363	2.99 × 10^−169^	F29	29.200	6.70 × 10^−136^	F6	21.473	1.30 × 10^−107^
F22	40.352	3.20 × 10^−169^	F26	28.523	1.27 × 10^−133^	F16	21.235	1.19 × 10^−106^
F18	40.205	7.90 × 10^−169^	F31	28.232	1.25 × 10^−132^	F12	20.857	4.10 × 10^−105^
F24	39.484	7.09 × 10^−167^	F23	27.416	8.11 × 10^−130^	F11	20.252	1.26 × 10^−102^
F4	38.114	4.23 × 10^−163^	F2	27.251	3.05 × 10^−129^	F25	20.159	3.08 × 10^−102^
F32	37.893	1.77 × 10^−162^	F17	26.437	2.25 × 10^−126^	F19	20.015	1.23 × 10^−101^
F15	37.379	4.96 × 10^−161^	F21	25.512	4.77 × 10^−123^	F8	17.615	2.84 × 10^−91^
F7	36.586	9.06 × 10^−159^	F14	25.227	5.23 × 10^−122^	F9	13.014	2.74 × 10^−69^
F33	34.914	6.93 × 10^−154^	F28	24.841	1.37 × 10^−120^	F30	12.261	2.18 × 10^−65^
F13	31.215	1.73 × 10^−142^	F20	24.781	2.29 × 10^−120^	F5	10.212	2.65 × 10^−54^
F1	30.873	2.17 × 10^−141^	F10	24.178	4.06 × 10^−118^	F3	7.398	6.82 × 10^−38^

**Table 6:** Correlations between the front and side face observations of the same soft face traits.

ID	Pearson’ *r* Correlation Coefficient	
F32	0.887	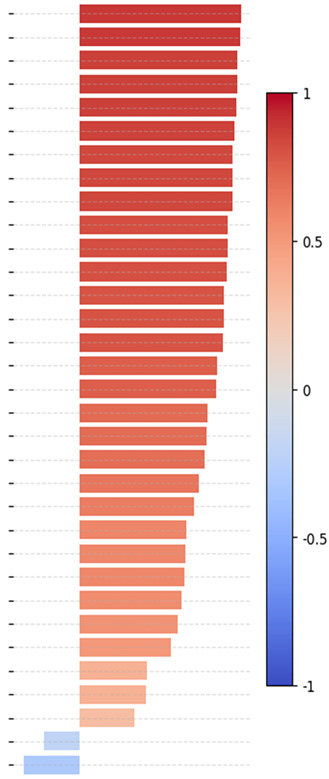
F22	0.886	
F15	0.867	
F7	0.867	
F27	0.863	
F33	0.852	
F4	0.843	
F24	0.843	
F19	0.840	
F13	0.818	
F18	0.817	
F8	0.812	
F6	0.797	
F31	0.794	
F30	0.790	
F21	0.757	
F10	0.750	
F26	0.704	
F20	0.697	
F28	0.688	
F29	0.659	
F5	0.632	
F14	0.590	
F16	0.583	
F9	0.579	
F23	0.561	
F25	0.539	
F11	0.504	
F17	0.371	
F1	0.368	
F3	0.302	
F12	−0.196	
F2	−0.305	

**Table 7:** Accuracy and precision of both traditional models and models augmented by soft face traits.

Approach	Accuracy (SVM)	Precision (SVM)	Soft Trait Weight Coefficient
ResNet-50	62.00%	56.53%	-
VGG-16	62.00%	64.80%	-
VGG-16 + soft_traits_33	66.00% 68.00%	69.38% 71.35%	3.5 5.5
ResNet-50 + soft_traits_33	72.00%	73.70%	-
VGG-16 + ResNet-50 + soft_traits_33	66.00% 68.00%	69.95% 70.48%	3.5 5.5
VGG-16 + soft_traits_33 + global_traits_6	68.00%	70.48%	5.5
**ResNet-50 + soft_traits_33 + global_traits_6**	**84.00%** **85.00%**	**79.47%** **81.13%**	**2.5** **3.5**

**Table 8:** The CMC and ROC scores of the traditional and augmented approaches.

Approach	Identification			Verification	
	Average Sum Match Scores Up to Rank				
	=1	=5	=10	AUC	EER
ResNet-50	0.570	0.870	0.940	0.963	0.107
VGG-16	0.570	0.790	0.870	0.944	0.150
ResNet-50 + soft33	0.640	0.910	**0.970**	0.976	0.075
VGG-16 + soft33	0.570	0.790	0.900	0.950	0.129
**ResNet-50 + soft33 + global6**	**0.810**	**0.930**	**0.970**	**0.986**	**0.062**

**Table 9:** Comparison between our study and previous studies associated with side face recognition.

Ref.	Dataset	Hard Features Extractor	Soft Biometrics	Features Fusion	Face View		Accuracy
					Training	Testing	
[13]	UT-DOOR, CMU Multi-PIE	PCA, LDA, LBP, and HOG	No	No	Side	Side id & verification	96.7%, 92.7%
[16]	XM2VTSDB	No	33 comparative face traits	No	Side	Side id	96.0%
[17]	FERET color dataset	No	3 patches for (hair, skin, and cloths colors)	No	Front	Side re-id	unknown
Ours	CMU Multi-PIE	ResNet-50, VGG-16	New proposed 33 face soft traits and 6 global soft traits	Yes	Front	Zero-shot Side id & verification	85.0%

## Data Availability

The dataset used in this article were CMU Multi-PIE. For details, please refer to [11,13,25,26].
